# Optimization of Human Corneal Endothelial Cells for Culture: The Removal of Corneal Stromal Fibroblast Contamination Using Magnetic Cell Separation

**DOI:** 10.1155/2012/601302

**Published:** 2012-01-12

**Authors:** Gary S. L. Peh, Man-Xin Lee, Fei-Yi Wu, Kah-Peng Toh, Deepashree Balehosur, Jodhbir S. Mehta

**Affiliations:** ^1^Singapore Eye Research Institute, Singapore 168751; ^2^Singapore National Eye Centre, Singapore 168751; ^3^Duke-NUS Graduate Medical School Singapore, Singapore 169857; ^4^Yong Loo Lin School of Medicine, National University of Singapore, Singapore 119228

## Abstract

The culture of human corneal endothelial cells (CECs) is critical for the development of suitable graft alternative on biodegradable material, specifically for endothelial keratoplasty, which can potentially alleviate the global shortage of transplant-grade donor corneas available. However, the propagation of slow proliferative CECs *in vitro* can be hindered by rapid growing stromal corneal fibroblasts (CSFs) that may be coisolated in some cases. The purpose of this study was to evaluate a strategy using magnetic cell separation (MACS) technique to deplete the contaminating CSFs from CEC cultures using antifibroblast magnetic microbeads. Separated “labeled” and “flow-through” cell fractions were collected separately, cultured, and morphologically assessed. Cells from the “flow-through” fraction displayed compact polygonal morphology and expressed Na^+^/K^+^ATPase indicative of corneal endothelial cells, whilst cells from the “labeled” fraction were mostly elongated and fibroblastic. A separation efficacy of 96.88% was observed. Hence, MACS technique can be useful in the depletion of contaminating CSFs from within a culture of CECs.

## 1. Introduction

The inner monolayer of the human cornea—the corneal endothelium (CE)—functions both as a barrier and a pump and plays a critical role in the regulation of corneal hydration. The CE layer prevents excessive fluids from entering the glycosaminoglycan-rich stromal layer while actively pumping fluid out to prevent corneal edema [1–3]. This maintains corneal thickness and keeps the corneal transparent [3, 4]. Cells of the CE do not have the capacity to undergo functional regeneration *in vivo *[5–7]. Hence, when corneal endothelial cell loss occurs, the existing cells spread out to maintain functional integrity of the CE. However, a critical threshold must be maintained to preserve corneal clarity. If endothelial dysfunction develops, there will be an inability to efficiently pump fluid out of the stroma, resulting in stromal and epithelial edema, loss of corneal clarity, and visual acuity [4], which will eventually lead to corneal blindness—a situation where the retina is normal but the cornea becomes edematous. This is the second leading cause of visual blindness worldwide [8]. Restoration of vision in these situations is only possible by replacing the dysfunctional CE layer with healthy donor CE through corneal transplantation.

There is a global shortage of transplant-grade donor corneal tissues, and this greatly restricts the number of corneal transplantation performed yearly [9, 10]. Hence, significant efforts have been garnered for the development of tissue-engineered cultured endothelial cells that may potentially circumvent the shortage of transplant-grade donor corneas [11]. To facilitate the development of a tissue-engineered endothelium, the ability to cultivate the human corneal endothelial cells (CECs) in an *in vitro *culture system is critical [12, 13].

The isolation and cultivation methods for the expansion of CECs evolved over the years and varied greatly between laboratories [14–20]. The current CECs isolated protocol used was adapted from a two-step “peel-and-digest” approach [21], using cadaveric research-grade donor corneas, whereby Descemet's membrane (DM), together with the corneal endothelium layer, is peeled off from the donor cornea before being subjected to enzymatic digestion using collagenase. However, this technique may result in the coisolation of contaminating stromal keratocytes in cases where the DM layer is difficult to peel and requires lengthy manipulation [8]. In our experience, this is especially so in donor corneas obtained from very young donors (unpublished observations). In these cases, a thin piece of posterior stroma can be partially torn off during the prolonged manipulation process and remains inconspicuously adhered to the DM-endothelial layer. Enzymatic dissociation to isolate the CECs from the DM layer will also release the undesired stromal keratocytes into the culture system. Subsequent exposure to complex serum-supplemented medium required for the culture and expansion of CECs will inevitably turn the contaminating stromal keratocytes into rapid growing stromal fibroblasts [22]. In isolations of primary CECs with fibroblastic contamination, we and others have found that the overgrowth of corneal stromal fibroblasts (CSFs) usually become apparent within 4 to 5 days of culture and will outgrow the less proliferative CECs within 7 days in culture [8, 23]. This is detrimental to the affected CEC cultures which generally take between 14 to 21 days to establish and will negatively impact on the development of tissue-engineered constructs where pure populations of cultivated functional CECs are required [8]. Furthermore, the consequence of fibroblastic contamination in these cultures will interfere with the critical barrier and pump function of the cultivated CECs [24]. Hence, there lies the need for methods to eliminate the contaminating CSFs.

Magnetic cell separation (MACS) technique using magnetic particles at both the micro- and nanoscale have been well described as standard tools for the isolation, purification, or separation of defined subset of cells, based on their specific cell-surface antigenic expression, in modern cell biology and immunology [25, 26]. The present study is aimed to evaluate the feasibility of using MACS technology to deplete contaminating CSFs from expanding CECs.

## 2. Materials and Methods

### 2.1. Isolation and Growth of Human Corneal Endothelial Cells

A total of three pairs research-grade corneoscleral tissues from cadaver human donors considered unsuitable for transplantation were obtained from Lions Eye Institute for Transplant and Research (Tampa, FL, USA). These research corneas, from donors between 24 to 33 years, were preserved and transported in Optisol-GS at 4°C and processed between 7 to 12 days from preservation (Table 1). Primary CECs were isolated using a modified two-step, peel-and-digest method. After three 15-minute washes in a PBS buffered antibiotic/antimycotic solution, the DM together with the corneal endothelial cells, anterior to Schwalbe's line, within a 9.0 mm diameter, was carefully peeled off from the stroma under a dissecting stereomicroscope. Paired DM-endothelial layer obtained were pooled and treated with collagenase (2 mg/mL; Roche, Mannheim, Germany) for at least 2 hours. The resultant CEC clusters were further dissociated into smaller clumps using TrypLE Express (TE; Invitrogen, Carlsbad, CA, USA) for approximately 5 minutes. Isolated CECs were cultured on FnC mixture (United States Biologicals, Swampscott, MA, USA) coated culture plates, in F99 medium (a 1 : 1 mixture of Ham's F12 (Invitrogen) and M199 (Invitrogen) media), supplemented with 5% fetal bovine serum (Invitrogen), 20 *μ*g/mL ascorbic acid (Sigma, St. Louis, MO, USA), 1x insulin-transferrin-selenium (Invitrogen), 1x antibiotic/antimycotic (Invitrogen), and 10 ng/mL of basic fibroblast growth factor (bFGF, R&D Systems, Minneapolis, MN, USA). After the primary cultures of CECs reached confluency at P0, as well as in subsequent passages, cells were dissociated using TE and subcultured on FnC-coated culture wares at a plating density of ~7,500 cells/cm^2^. For this study, CECs were utilized at the third passage. All incubation and cell culture were carried out in a humidified incubator (Binder, Bohemia, NY, USA) at 37°C containing 5% CO_2_.

### 2.2. Isolation and Growth of Human Corneal Stromal Fibroblast

After the DM-endothelial layer has been peeled from the stroma, an 8.5 mm stroma button was obtained by trephination. The corneal epithelial layer was carefully scraped off using a scalpel blade. Stroma buttons (*n* = 3) were washed twice in a PBS-buffered antibiotic/antimycotic solution and enzymatically digested in collagenase overnight. The following day, stromal keratocytes released from within the stroma button were briefly washed twice with PBS, seeded onto cell culture flask coated with FNC coating mixture, and cultured in F99 medium. The exposure to a serum-supplemented medium transformed the corneal stromal keratocytes into CSFs. Culture medium was refreshed every two days, and confluent fibroblast cultures were passaged using TrypLE Express in a 1 : 5 split ratio. For this study, CSFs were utilized at the eighth passage.

### 2.3. Labeling Corneal Stromal Fibroblast with CMFDA CellTracker Green

In order to assess the efficacy of the cell separation, cultured CSFs (*n* = 3) were prelabeled with CellTracker Green, 5-chloromethylfluorescein diacetate (CMFDA; Invitrogen) as described [27]. Briefly, CSFs were incubated in a serum-free medium containing 5 *μ*M of CMFDA dye for 30 minutes at 37°C. Subsequently, the CMFDA dye containing medium was replaced with fresh serum-free medium for another 30 minutes at 37°C to allow the cellular metabolites of CMFDA to be removed, which transforms the colorless and nonfluorescent CMFDA into a brightly fluorescent green product.

### 2.4. Magnetic Cell Separation and Determination of MACS Efficacy

Separate culture of CMFDA labeled CSFs and CECs was each trypsinized using TrypLE Express (Invitrogen, Carlsbad, CA, USA) and counted using a hemocytometer. Subsequently, the CMFDA-labeled CSFs and CECs were mixed in this study at a 1 : 1 ratio to achieve an approximated 50% mixture of CSFs and CECs within a suspension (*n* = 3). The cell mixture was then washed once in PBS and immediately resuspended in MACS buffer (PBS containing 2 mM EDTA, and 0.5% BSA) and incubated with antifibroblast magnetic microbeads (Miltenyi Biotech) for 15 minutes at room temperature. This was followed by the resuspension of the cell mixture in 500 *μ*L of MACS buffer and applying it to the MS column attached to the Mini-MACS separator (Miltenyi Biotech). Subsequently, the column was washed twice (500 *μ*L each) with MACS buffer. The total effluent was collected as the “flow-through” fraction. The MS column was then removed from the magnet, and the magnetically retained cells within the column were flushed out in 1 mL of MACS buffer by the means of a plunger. This effluent was collected as the “labeled fraction.” The “labeled” fraction, “flow-through” fraction, and an aliquot of the unsorted cell mixture were collected separately and subcultured on FnC-coated culture ware in F99 medium for further analysis (*n* = 3). The efficacy of MACS to deplete the CSFs from the heterogeneous population of CSFs and CECs, mixed in a 1 : 1 ratio, can be determined by the number of CMFDA-labeled cells found in the “flow-through” fraction 1 day after-separation, using the formula:

(1) $$E=\left(1-\frac{\text{\%}\;{\text{Fibroblast}}_{\text{post-separation}}}{\text{\%}\;{\text{Fibroblast}}_{\text{pre-separation}}}\right)\times 100\%.$$

### 2.5. Antibodies and Immunofluorescence

At Day 3, separated cell fractions cultured on glass slides (*n* = 3) were fixed on the third day after-separation in 100% ice-cold ethanol for 5 minutes. The ethanol-fixed cells were immersed in a PBS block solution containing 10% normal goat serum. Following this, the samples were incubated with primary and, subsequently, secondary antibody (in the dark), each for 1 hour at room temperature. Between each incubation steps, samples were washed twice with PBS. Labeled cells were mounted onto coverslips in Vectorshield mounting medium containing DAPI (Vector Laboratories). The following primary antibody used was mouse IgG_1_ anti-Na^+^/K^+^ATPase *α*1 (5 *μ*g/mL; Santa Cruz Biotechnology). The secondary antibody used was Alexa Fluor 546 goat anti-mouse IgG (2 *μ*g/mL; Invitrogen). Negative controls were cells incubated with an anti-mouse IgG_1_ isotype control (5 *μ*g/mL; BioLegends) in place of the primary antibody. Fluorescent images of cells were examined using a Zeiss Axioplan 2 fluorescence microscope (Carl Zeiss, Germany). At least 250 nuclei were analyzed from five randomly selected fields per experiment (*n* = 3).

### 2.6. Morphometry Analysis of Cellular Circularity

The healthy corneal endothelial cells of the human corneal endothelium are mostly hexagonal in shape [28, 29], and one of the morphological characteristic of isolated human corneal endothelial cells *in vitro *is the maintenance of the unique polygonal cellular structure in culture [8, 30, 31]. Cell circularity can be determined using the formula: Circularity = Perimeter^2^/(4*π* × Area), which quantifies the roundness of the cell assessed, where a value approaching 1.0 is equivalent to a cell with a cellular profile nearing circularity. Hence, the polygonal CECs will have a profile closer to 1.0 compared to the long and spindle-like CSFs. Digital micrographs of cultured cells from separated “labeled” and “flow-through” fractions were taken on the third day after-separation. The area and perimeter of randomly selected cells were analyzed with Image J software (NIH, Bethesda, MD, USA). At least 100 cells from each of the “labeled” and “flow-through” fraction (*n* = 3) were analyzed.

### 2.7. Statistical Analysis

All numerical data obtained were expressed as a mean ± standard deviation. Comparison of cell circularity was statistically analyzed using Mann-Whitney *U* test (SPSS Statistics 17.0, IBM, Chicago, IL, USA). Values were deemed to be significant when a significance level with a *P* value of less than 0.05 was achieved.

## 3. Results

### 3.1. Culture of Corneal Endothelial Cells

**Figure 1:**
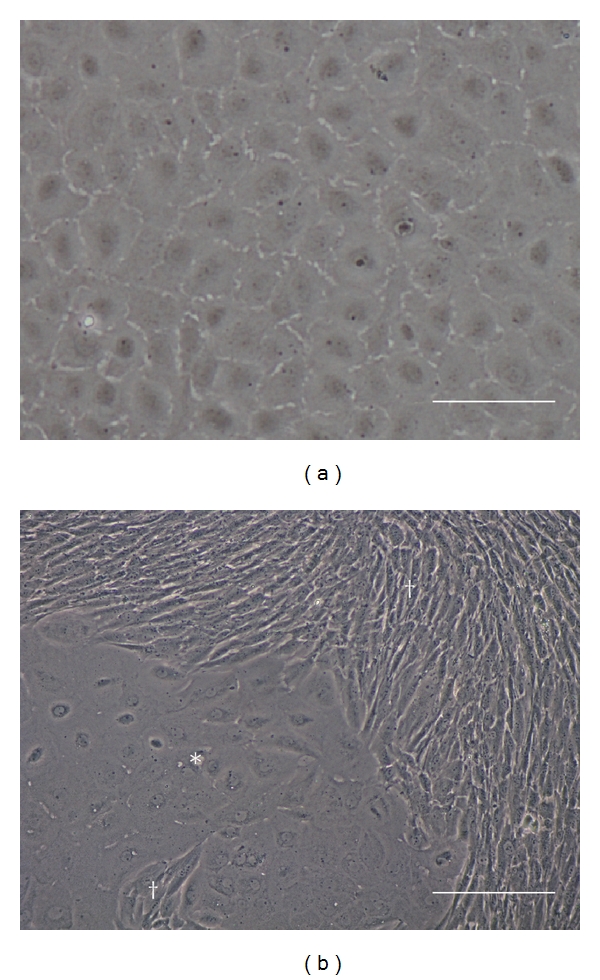
(a) Representative micrograph of a confluent homogeneous monolayer of successfully isolated human corneal endothelial cells at Day 14. (b) Representative micrograph of a failed isolation attempt resulting in stromal contamination at Day 7. The cellular boundary between the polygonal corneal endothelial cells (*) and the confluent elongated corneal stromal fibroblasts (^†^) can be clearly defined (scale bars = 100 *μ*m).

An established monolayer of primary CECs isolated from the human corneal endothelium takes between 14 to 21 days and can be determined morphologically by their unique polygonal cellular structure (Figure 1(a)). In comparison, an example of a CECs culture contaminated with the CSFs contains heterogeneous cell populations with two distinct cell types, the polygonal CECs (*) and the elongated CSF (^†^) (Figure 1(b)).

### 3.2. CMFDA-Labeled CSFs and Mixture with Unlabeled CECs

**Figure 2:**
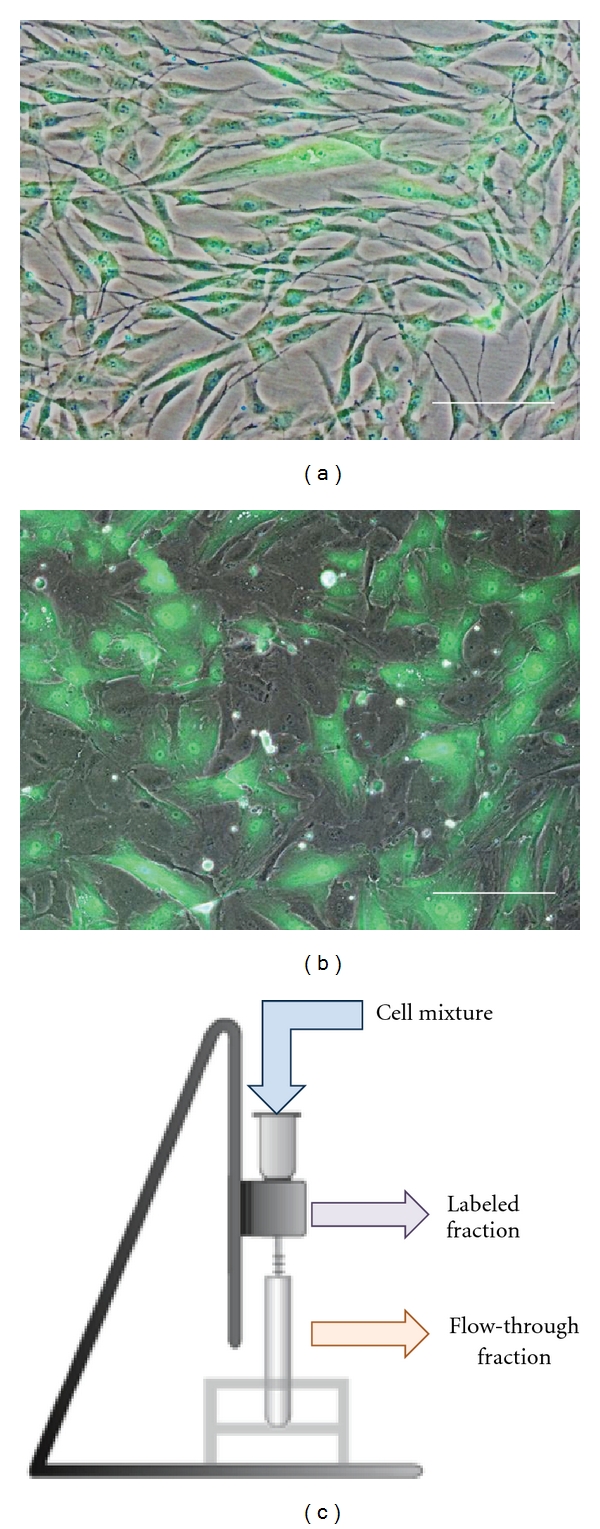
(a) Composite phase contrast and fluorescent picture of corneal stromal fibroblast labeled experimentally with CMFDA CellTracker Green dye. (b) Culture of CMFDA labeled corneal stromal fibroblast with human corneal endothelial cells 24 hours after mixing at a 1 : 1 ratio. (c) Schematic of the MACS setup used in the study (scale bar = 100 *μ*m).

The CMFDA-labeled CSFs maintained bright green fluorescent after 3 days in culture (Figure 2(a)). However, due to the rapidly expanding CSFs, the level of green fluorescent decreases exponentially and could not be detected by fluorescent microscopy by the seventh day in culture (results not shown). The CMFDA-labeled CSFs and unlabeled CECs were experimentally mixed at a 1 : 1 ratio and assessed 24 hours after the cell mixture were and left to adhere. Composite phase contrast and fluorescent image (Figure 2(b)) showed that CSFs constituted to approximately 55.38%  ± 7.16% of the adhered mixed culture.

### 3.3. Separation Efficiency of Mixed CSFs and CECs Cultures

The experimentally mixed cultures of CSFs and CECs were subsequently tagged with antifibroblast magnetic microbeads and subjected to the magnetic separation through the MS column placed within a magnetic field and separated into the “labeled” and “flow-through” fraction as depicted in the schematic (Figure 2(c)).

**Figure 3:**
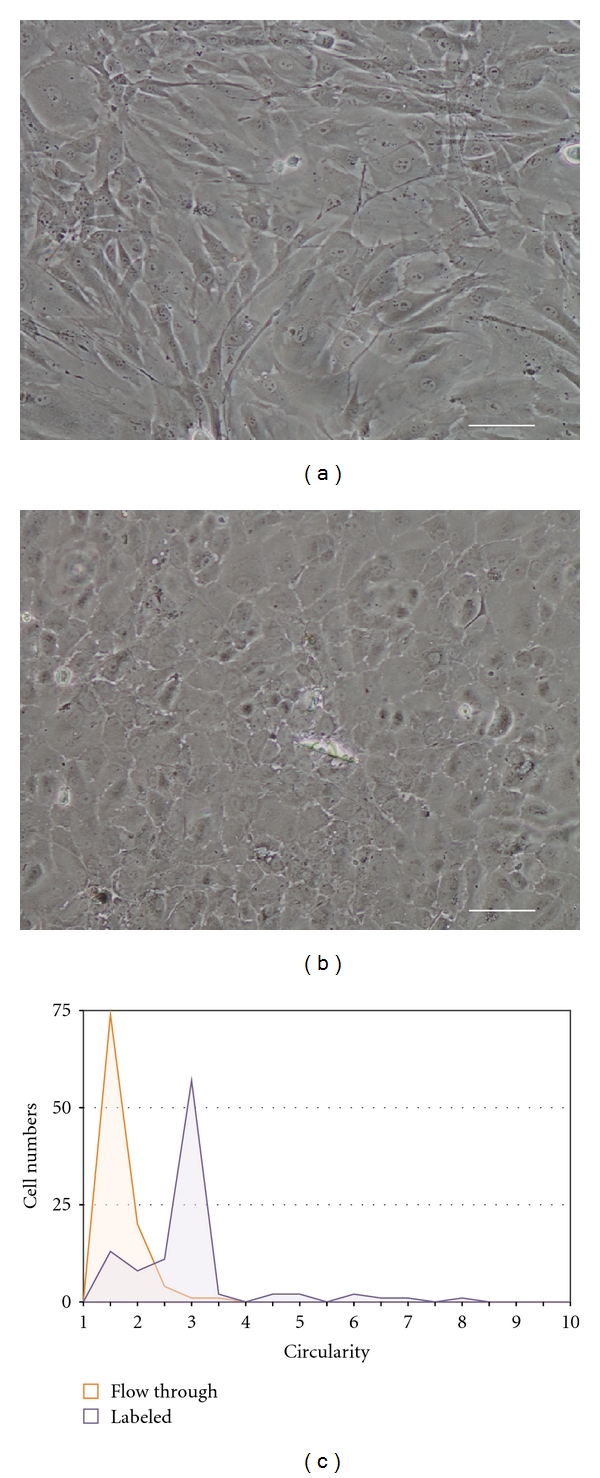
Following MACS of experimentally mixed human corneal endothelial cells and corneal stromal fibroblast using antifibroblast magnetic microbeads, cell fractions were subcultured as (a) the antifibroblast magnetic microbeads “labeled” fraction and (b) unlabeled “flow-through” fraction, 3 days after sorting. (Scale bar = 100 *μ*m.) (c) A frequency histogram depicting the circularity of randomly selected cells in the “labeled” and the “flow-through” fractions. At least 100 cells from each fraction were counted.

The MAC-separated cells were left to adhere for at least one day to enable the cells to establish their morphology, and postseparation assessment of cultured fluorescent cells from both the “label” (Figure 3(a)) and “flow-through” (Figure 3(b)) fractions of the separated mixtures was performed to determine the efficacy of separation. Approximately 81.66%  ± 3.46% of the cells from the “labeled” fraction were fluorescent, compared to 1.73%  ± 1.09% from the “flow-through” fraction following separation. The efficacy of separation was deemed to be approximately 96.88%  ± 3.50% (Table 2).

### 3.4. Morphometric Assessment of Cellular Circularity

The morphometric assessment of cultured cells from the “labeled” and “flow-through” fractions of separated mixtures was performed on the third day after-separation. At least 100 cells from each of the “labeled” and “flow-through” fraction were randomly selected and assessed for their cellular circularity. The morphometric assessment showed that cultured cells from the “flow-through” fraction were “rounder” with a circularity profile of 1.42 ± 0.35, indicative of the polygonal CECs, compared to the “labeled” fraction containing cells of significantly mixed circularity profile of 2.61 ± 1.10 (*P* < 0.001; Figure 3(c); Table 2).

### 3.5. Expression of Ion Channel Na^+^/K^+^ATPase after MACS

**Figure 4:**
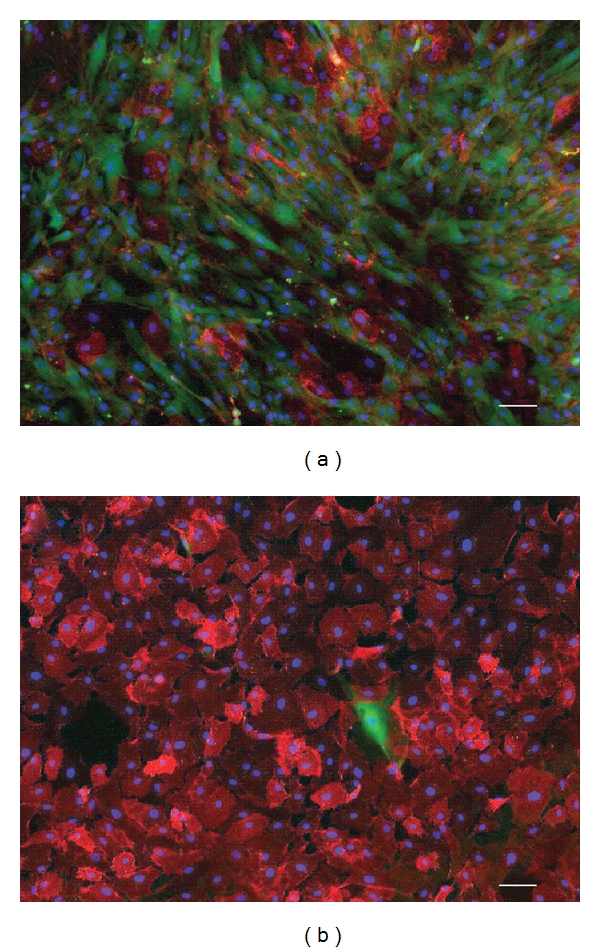
Composite fluorescent micrograph images of CMFDA (green) and Na^+^/K^+^ATPase pumps (red) of cultured cells established from (a) the “labeled” fraction and (b) the “flow-through” fraction. Cell nuclei were counterstained with DAPI in blue. (Scale bar = 100 *μ*m.)

Majority of the cells in the “labeled” fraction were found to be CMFDA positive with 12.3%  ± 0.2% of the cells that strongly expressed Na^+^/K^+^ATPase (Figure 4(a)). Conversely, in the “flow-through” fraction, approximately 98.66%  ± 0.1% of the cells were Na^+^/K^+^ATPase positive (Figure 4(b)), displaying a ubiquitous staining pattern.

## 4. Discussion

The capacity to propagate primary CECs *in vitro *is critical for the development of alternative donor graft material suitable for corneal transplantation. However, this process can be hindered by the coisolation of the stromal keratocytes located within the adjacent layer of the cornea. These contaminating corneal stromal keratocytes will transform into fast growing CSFs in the presence of complex culture medium containing serum, which is crucial for the extended culture of CECs. Rapid growth of contaminating CSFs will outgrow and impede the growth of CECs and will hinder the development of tissue-engineered graft alternative where a pure population of cultivated CECs is required, and the use of CSFs-contaminated CECs will impede the functional integrity of the engineered graft substitute rending the construct ineffective. The two-step “peel-and-digest” method used currently involves peeling of the DM layer within an approximated 9.0 mm diameter to decrease chances of coisolating noncorneal endothelial cells that lie beyond the posterior Schwalbe's line, which includes the trabecular meshworks and the underlying cells with the potential to turn into fibroblasts. This restricts the isolation of the most peripheral region of the corneal endothelium layer, which has been shown to contain endothelial cells that are more proliferative [32, 33]. Moreover, the human cornea has a diameter of approximately 11.5 mm. If a larger area of the DM endothelial layer can be removed during the isolation process with a solution to restrict potential fibroblastic overgrowth, the final yield of the isolated CECs for culture can be significantly increased. For example, the difference in the amount of CECs isolated between taking an area of 9.0 mm and an area of 11.0 mm with a corneal endothelial density cell count of 2,000 cells per mm^2^ equates to a 33.06% difference or approximately 63,000 cells from a single donor cornea.

Engelmann and colleagues previously reported that fibroblastic contamination could be eliminated from corneal endothelial cell culture using a D-valine supplemented medium that is L-valine-free [23], based on the rationale that fibroblasts lack the D-amino-acid oxidase required for the conversion of D-valine to L-valine [34]. However, complete elimination of L-valine and associated mitogens known to promote the growth of fibroblast from a culture medium is cumbersome. For example, the serum used in the medium was predialyzed for 24 hours three times against a 50-fold volume of tissue culture grade water, and once against a 2-to 3-fold volume of L-valine-free medium [23]. Furthermore, the dialysed serum was processed to be free of potent fibroblasts mitogens such as fibroblast growth factor and platelet-derived growth factor before they were used in the supplementation of the selection medium [23]. For laboratories using different culture medium in the cultivation of CECs, these processes to remove L-valine, as well as associated mitogens, must also be applied to the other complex supplementation used in the medium, such as bovine pituitary extract [35].

Cell separation using MACS technology can be performed using two approaches, namely, positive selection in which the antigens on the surface of the cell of interest are targeted by specific antibody coupled with the magnetic microbeads and negative depletion where microbeads used target the unwanted cells and leave the cell of interest untouched. However, since antigen specific to CECs has yet been identified, a positive selection approach cannot be utilized. Therefore, in this study, we adopted the negative depletion method to purify the cultured CECs from the contaminating CSFs within an experimental setting. This method offers a relatively simple alternate option to eliminate fibroblastic contamination that may occur following the isolation and propagation of CECs. We and others showed that separated cells from both the “labeled” and “flow-through” fractions could be subcultured, which enabled downstream morphometric assessment and characterization [26, 36]. Cultured cells from the “labeled” fraction were found to contain 81.66%  ± 3.46% fibroblasts as shown by their long and spindle-like morphology and green fluorescent CMFDA dye. In this fraction, the remaining cells, approximately 12.3%  ± 0.2% as judged by the expression of Na^+^/K^+^ATPase, were the CECs, which were most likely trapped within the magnetized column during the separation process, and could only be recovered at the end of the procedure together with the remaining labeled fibroblasts when they were flushed out of the MS column. Morphometric assessment of cellular circularity reflected the above observation with a significantly mixed cellular circularity profile detected in the “labeled” fraction. On the other hand, cultured cells from the “flow-through” fraction were more homogeneous in terms of their circularity. Hence, for our application to separate CECs from a mix culture of CECs and CSFs, a negative selection approach appeared to be more efficient as compared to positive selection.

Activity of Na^+^/K^+^ATPases is associated with the liquid pump function of the corneal and is critical for the proper physiological control of corneal thickness by the corneal endothelium [1, 37, 38]. The expression of the Na^+^/K^+^ATPase not only showed the viability of the separated CECs following MACS, it also suggests that the CECs remained unaltered in their functionality.

From the expression of CMFDA dye, approximately 1.73% of the cells in the “flow through” were deemed to be the CSFs that have escaped the separation procedure. Based on these observations, the efficacy of MACS using the antifibroblast magnetic microbeads was deemed to be over 96.88% in the present experimental setting, where the CECs were mixed with the CSFs at a 1 : 1 ratio. However, it should be noted that a minute amount of viable CSFs that escaped the separation process, given enough time, will be sufficient to overtake a culture of generally slow-growing CECs. Application of the “flow-through” fraction through another magnetized MACS column may reduce the numbers of contaminating fibroblast further but will also increase the numbers of CECs that become trapped within the column. Alternatively, the cultured “flow-through” fraction can be closely monitored following MAC separation and regions of rapid cell growth with spindly fibroblastic morphology can be immediately scrapped off manually under the dissecting microscope. The elimination of such fibroblastic contaminants via manual scraping is more plausible when the contaminated regions are small.

## 5. Conclusion

In conclusion, our results demonstrated that current MACS technology using the antifibroblast magnetic microbeads provides a simple alternative method to deplete majority of the contaminating CSFs in situations where precious cultures of CECs were found to be contaminated with rapid-growing CSFs. Further development of this technology is required to enable the complete depletion of all contaminating CSFs, and this may involve the development of multiple magnetic microbeads tagged antibodies detecting different cell-surface antigens expressed specifically by CSFs.

## Figures and Tables

**Table 1:** Donor information.

Serial Number	Age	Sex	Days to Culture	Cause of Death
01	33	M	7	Acute Cardiac Crisis
02	24	F	12	Acute Cardiac Crisis
03	28	M	9	Overdose

Cultures of human corneal endothelial cells were established from donors aged 24 year-old to 33 year-old. Days taken from death of donor to the initiation of corneal endothelial cell culture ranged from 7 days to 12 days with a median of 9 days.

**Table 2:** Efficacy of MACS.

Fraction	CMFDA Positive Cells (%)	Circularity Index
Unsorted Mixture	55.38 ± 7.16	Not Measured
“Labeled”	81.66 ± 3.46	2.61 ± 1.10*
“Flow-Through”	1.73 ± 1.09	1.42 ± 0.35*

For the comparison of cell circularity index, significance was achieved between the “labeled” and “flow-through” fraction (*z* = −9.62  *P < 0.001). The overall sorting efficacy of MACS was calculated as 96.88%  ± 3.50%.
